# Characteristics and Risk Factors of Hospitalized and Nonhospitalized COVID-19 Patients, Atlanta, Georgia, USA, March–April 2020

**DOI:** 10.3201/eid2704.204709

**Published:** 2021-04

**Authors:** Kristen Pettrone, Eleanor Burnett, Ruth Link-Gelles, Sarah C. Haight, Caroline Schrodt, Lucinda England, Danica J. Gomes, Mays Shamout, Kevin O’Laughlin, Anne Kimball, Erin F. Blau, Chandresh N. Ladva, Christine M. Szablewski, Melissa Tobin-D’Angelo, Nadine Oosmanally, Cherie Drenzek, Sean D. Browning, Beau B. Bruce, Juliana da Silva, Jeremy A.W. Gold, Brendan R. Jackson, Sapna Bamrah Morris, Pavithra Natarajan, Robyn Neblett Fanfair, Priti R. Patel, Jessica Rogers-Brown, John Rossow, Karen K. Wong, David J. Murphy, James M. Blum, Julie Hollberg, Benjamin Lefkove, Frank W. Brown, Tom Shimabukuro, Claire M. Midgley, Jacqueline E. Tate, Marie E. Killerby

**Affiliations:** Centers for Disease Control and Prevention, Atlanta, Georgia, USA (K. Pettrone, E. Burnett, R. Link-Gelles, S.C. Haight, C. Schrodt, L. England, D.J. Gomes, M. Shamout, K. O’Laughlin, A. Kimball, E.F. Blau, C.N. Ladva, C.M. Szablewski, S.D. Browning, B.B. Bruce, J. da Silva, J.A.W. Gold, B.R. Jackson, S.B. Morris, P. Natarajan, R.N. Fanfair, P.R. Patel, J. Rogers-Brown, J. Rossow, K.K. Wong, T. Shimabukuro, C.M. Midgely, J.E. Tate, M.E. Killerby);; Commissioned Corps of the U.S. Public Health Service, Rockville, Maryland, USA (R. Link-Gelles, R.N. Fanfair, J. Rossow, J.E. Tate);; Georgia Department of Public Health, Atlanta (C.M. Szablewski, M. Tobin-D’Angelo, N. Oosmanally, C. Drenzek);; Emory University School of Medicine, Atlanta (D.J. Murphy, J.M. Blum, J. Hollberg, F.W. Brown);; Emory Decatur Hospital, Decatur, Georgia, USA (B. Lefkove, F.W. Brown)

**Keywords:** COVID-19, risk factors, care seeking, symptoms, age, concurrent conditions, respiratory infections, severe acute respiratory syndrome coronavirus 2, coronavirus disease, zoonoses, viruses, coronavirus, SARS-CoV-2, Atlanta, Georgia, United States

## Abstract

We compared the characteristics of hospitalized and nonhospitalized patients who had coronavirus disease in Atlanta, Georgia, USA. We found that risk for hospitalization increased with a patient’s age and number of concurrent conditions. We also found a potential association between hospitalization and high hemoglobin A1c levels in persons with diabetes.

Information about care-seeking behavior, symptom duration, and risk factors for progression to severe illness in nonhospitalized patients with coronavirus disease (COVID-19) aids in resource planning, disease identification, risk stratification, and clinical management of nonhospitalized patients (*1*–*6*). We built on a previous analysis comparing hospitalized and nonhospitalized COVID-19 patients, which found that hospitalized patients were more likely to be >65 years of age, men, Black, diabetic, or obese (*7*). We describe symptom patterns, duration of illness, and care-seeking behavior among nonhospitalized patients and explore the relationships between hospitalization and the number, control, and interaction of concurrent medical conditions and age. We defined control as how well a disease is managed in the patient, as measured by hemoglobin A1c levels in diabetics, number of classes of hypertension medication being taken by patients with hypertension, and BMI among patients with obesity.

## The Study

We enrolled hospitalized and nonhospitalized patients >18 years of age with laboratory-confirmed COVID-19 (defined as a positive real-time reverse transcription PCR result for severe acute respiratory syndrome coronavirus 2) treated at 6 acute care hospitals and outpatient clinics affiliated with a single academic hospital system in the Atlanta, Georgia, USA, metropolitan area during March 1–April 7, 2020, as previously described (*7*). In this investigation, we compared characteristics and symptoms of hospitalized and nonhospitalized persons using χ^2^, Fisher exact, or *t*-test as appropriate.

We conducted univariable and multivariable logistic regression analyses to explore the associations between age group and number of underlying conditions on risk for hospitalization. We conducted separate analyses to model the associations and interactions of diabetes, hypertension, and obesity with hospitalization for COVID-19. We then used univariable and multivariable logistic regression to investigate whether the severity or control of concurrent conditions was associated with increased risk for hospitalization. We modeled the association between body mass index (BMI) and hospitalization. Further, we investigated whether use of multiple classes of hypertension medication was associated with hospitalization among patients with hypertension the association of elevated levels (>7%) of hemoglobin A1c (HbA1c) and hospitalization among patients with diabetes. This level was chosen because a value <7% is considered an indicator of adequate blood glucose control in patients with diabetes (*8*). Multivariable models were adjusted for characteristics previously associated with hospitalization in these populations (*7*) (Appendix).

We enrolled 311 nonhospitalized and 220 hospitalized patients in this study (Appendix Tables 1–3). We reviewed patient medical records and found that upper respiratory system symptoms including rhinorrhea, nasal congestion, and pharyngitis were more common among nonhospitalized patients than hospitalized patients (74% vs. 17%; p = 0.01). In contrast, hospitalized patients had dyspnea more frequently than did nonhospitalized patients (68% vs. 43%; p = 0.01) (Figure). Of 147 nonhospitalized patients with available information on symptom duration, 67 (46%) reported symptoms lasting >21 days.

**Figure:**
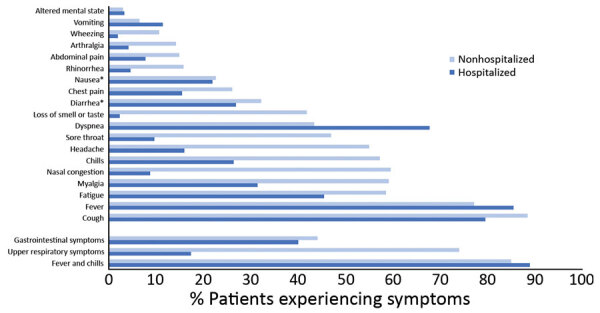
Symptoms of coronavirus disease among hospitalized and nonhospitalized patients, Atlanta, Georgia, USA, 2020. Gastrointestinal symptoms include vomiting, nausea, diarrhea, and abdominal pain. Upper respiratory symptoms include sore throat, rhinorrhea, and nasal congestion. *p>0.01.

Of 311 nonhospitalized patients, 135 (43%) had their first contact with the healthcare system for their COVID-19 illness on a telephone triage line, 23 (7%) at the emergency department, and 141 (45%) at an ambulatory care clinic (Table 1). Of nonhospitalized patients, 85% sought in-person care (i.e., ambulatory care, emergency department, or urgent care) a single time for their COVID-19 illness. A subset of 188 nonhospitalized patients had information in their medical records about all their COVID-19 healthcare visits. These 188 patients had 400 documented healthcare visits: 188 (47%) ambulatory care, 167 (42%) telehealth, 39 (10%) in-person emergency department, and 6 (2%) urgent care visits. Within this subset, 57% of visits among those patients with symptoms lasting >21 days were telehealth appointments; 56% of visits among those with symptoms <21 days were in-person primary care visits.

**Table 1:** Treatment settings of 311 nonhospitalized patients with coronavirus disease, Atlanta, Georgia, USA, 2020*

Treatment setting	Value
First interaction	
Ambulatory care	141 (45)
Telephone triage line	135 (43)
Emergency department	23 (7)
Other†	12 (4)
All interactions	
Ambulatory care	269 (87)
Telephone triage line	210 (68)
Emergency department	45 (15)
Other†	22 (7)
Median no. visits (IQR)	1 (1–1)
Ambulatory care	1 (1–1)
Telephone triage line	1 (1–2)
Emergency department	1 (1–1)

*Values are no. (%) patients except as indicated. IQR, interquartile range. †Includes retail health, telehealth, and urgent care.

Odds of hospitalization increased with advancing age (50–59 years of age, adjusted odds ratio [aOR] 2.1, 95% CI 0.7–6.6; 60–69 years, aOR 4.1, 95% CI 1.3–13.3; >70 years, aOR 9.2, 95% CI 2.7–31.0). The aOR of hospitalization demonstrated a dose-dependent relationship with number of concurrent conditions (1 condition, aOR 1.8, 95% CI 0.8–3.7; 2 conditions, aOR 2.3, 95% CI 1.1–4.8; >3 conditions, aOR 4.2, 95% CI 1.9–9.1) (Table 2).

**Table 2:** Risk factors for hospitalization among patients with coronavirus disease, Atlanta, Georgia, USA, 2020*

Characteristic	Hospitalized, no. (%)	Nonhospitalized, no. (%)	Crude OR (85% CI)	Adjusted OR (95% CI)
Age, y				
Total	220 (100)	311 (100)		
18–29	5 (2)	52 (17)	Referent	Referent†
30–39	24 (11)	79 (25)	3.0 (1.1–8.2)	1.4 (0.4–4.6)
40–49	36 (16)	54 (17)	6.3 (2.3–16.8)	3.0 (0.9–9.5)
50–59	41 (19)	63 (20)	6.4 (2.4–16.9)	2.1 (0.7–6.6)
60–69	56 (26)	41 (13)	13.9 (5.2–37.2)	4.1 (1.3–13.3)
>70	58 (26)	22 (7)	25.7 (9.2–71.4)	9.2 (2.7–31.0)
No. concurrent conditions				
Total	220 (100)	311 (100)		
0	21 (10)	122 (39)	Referent	Referent‡
1	48 (22)	80 (26)	3.5 (1.9–6.3)	1.8 (0.8–3.7)
2	71 (32)	68 (22)	6.0 (3.4–10.6)	2.3 (1.1–4.8)
>3	80 (36)	41 (13)	12.2 (6.6–22.4)	4.2 (1.9–9.1)
Hemoglobin A1c§				
Total	81 (100)	30 (100)		
<7%	17 (21)	17 (57)	Referent	Referent¶
>7%	38 (47)	7 (23)	3.3 (1.2–9.4)	4.1 (0.9–19.1)
Missing data	26 (32)	6 (20)		
Obesity				
Total	220 (100)	311 (100)		
<30	86 (39)	123 (40)	Referent	Referent#
30–34	65 (30)	52 (17)	1.8 (1.2–3.0)	2.6 (1.3–5.0)
35–40	34 (16)	26 (8)	1.9 (1.0–3.5)	2.2 (1.0–4.8)
>40	25 (11)	26 (8)	1.6 (0.8–2.9)	1.8 (0. 7–4.5)
Missing data	10 (5)	84 (27)		
No. classes of hypertension medications**				
Total	142 (100)	101 (100)		
0	20 (14)	13 (13)	Referent	Referent#
1	38 (27)	42 (42)	0.7 (0.3–1.6)	0.7 (0.3–2.0)
2	48 (34)	33 (33)	1.0 (0.5–2.4)	1.6 (0.6–4.5)
>3	36 (25)	13 (13)	1.9 (0.7–5.0)	1.8 (0.5–6.0)

*OR, odds ratio. †Adjusted for number of underlying conditions, race, sex, insurance, and smoking (including current or former smoking).  ‡Adjusted for age, race, sex, insurance, and smoking (including current or former smoking). §Among patients with diabetes. ¶Adjusted for age, race, sex, healthcare personnel status, and hypertension. #Adjusted for age, race, sex, healthcare personnel status, and diabetes. **Among patients with hypertension.

Among patients with hypertension, the odds of hospitalization demonstrated a possible dose-dependent increase among patients taking multiple classes of hypertension medications; however, precision of estimates was limited by small sample size (Table 2). Among patients with diabetes, those with a recent HbA1c score >7% had an increased risk for hospitalization (aOR 4.1, 95% CI 0.9–19.1); however, precision of estimates was limited by small sample size. Among obese patients (BMI >30), BMI was not associated with increasing odds of hospitalization (Table 2). In the multivariable analyses, we did not detect significant additive or multiplicative interaction between diabetes and obesity, hypertension and obesity, or hypertension and diabetes (Appendix Table 4).

## Conclusions

Symptoms lasting >21 days were common among nonhospitalized patients in this investigation; however, <20% of these patients had >1 in-person healthcare visit for COVID-19 during acute illness. These extended symptom durations, in conjunction with limited care-seeking behavior, suggest that many mildly ill COVID-19 patients can self-manage their symptoms. Because telemedicine was the second most common healthcare delivery method in our investigation, we hypothesize that it might have provided ongoing patient support and decreased the need for in-person healthcare visits (*9*). These findings can assist healthcare providers with anticipatory guidance for patients and caregivers and can inform decisions about allocation of resources for healthcare delivery.

We found that age and number of underlying conditions were associated with a dose-dependent increase in likelihood of hospitalization. Elderly COVID-19 patients frequently have multiple conditions that increase risk for hospitalization and serious infection (*10*). However, we did not find a significant additive or multiplicative interaction between the 3 most common underlying conditions among study participants: hypertension, diabetes, and obesity.

We hypothesized that degree of control of underlying conditions would affect risk for hospitalization. We found that the aORs for hospitalization were higher among patients with diabetes who had elevated mean levels of HbA1c and among patients with hypertension taking an increasing number of hypertension medications. Although not statistically significant, these findings may suggest an association between the management of concurrent conditions and COVID-19 disease severity. Despite obesity’s association with increased risk for severe illness and death from COVID-19 (*11*,*12*), we did not find an increasing risk for hospitalization with increasing BMI among persons with obesity. 

A limitation of our study is that, because of small sample sizes, our analyses might have lacked power to detect a significant association between degree of control of underlying conditions and hospitalization. In addition, our sample comprised patients at a single hospital system during a limited timeframe, and thus our results might not be generalizable to other populations. Because this hospital system prioritized certain persons (e.g., older patients, patients with underlying conditions, and healthcare personnel) for outpatient SARS-COV-2 testing, these persons might be overrepresented among the nonhospitalized patients in our sample. We were also not able to assess symptom resolution among all patients during the timeframe of this investigation and therefore might not have accounted for all follow-up healthcare visits for COVID-19.

In conclusion, although many nonhospitalized patients in this study reported symptoms lasting >21 days, most cases of COVID-19 among nonhospitalized patients were managed with a single ambulatory care visit and telehealth follow-up appointments. Patients of increasing age, with a greater number of underlying conditions, and with poor management of those conditions might be at higher risk for hospitalization and severe disease from COVID-19.

Appendix Further information on characteristics and risk factors of hospitalized and nonhospitalized COVID-19 patients, Atlanta, March–April 2020.
