# Supplementary material for: Characteristics and Risk Factors of Hospitalized and Nonhospitalized COVID-19 Patients, Atlanta, Georgia, USA, March–April 2020
Source: Emerg Infect Dis. 2021 Apr;27(4):1164–8. doi: 10.3201/eid2704.204709 (PMC8007327; doi:10.3201/eid2704.204709)
Supplement: Appendix — Further information on characteristics and risk factors of hospitalized and nonhospitalized COVID-19 patients, Atlanta, March–April 2020. [file 20-4709-Techapp-s1.pdf]

Article DOI: https://doi.org/10.3201/eid2704.204709

Characteristics and Risk Factors of Hospitalized and Nonhospitalized COVID-19 Patients, Atlanta, Georgia, USA, March–April 2020

Appendix

Methods

Data Collection

Hospitalized and nonhospitalized patients ≥18 years of age with laboratory-confirmed coronavirus disease (COVID-19) (defined as a positive real-time reverse transcription PCR for severe acute respiratory syndrome coronavirus 2 [SARS-CoV-2]) who were treated at 6 acute care hospitals and outpatient clinics affiliated with a single academic hospital system in the Atlanta metropolitan area were included in the study. Patients who were hospitalized for COVID-19 during March 1–30, 2020 (including those who stayed for observation or died in the emergency department) were sequentially selected from lists provided by the health system and reviewed over a 3-week period in April 2020.

Nonhospitalized patients were identified from the provided lists of patients ≥18 years of age who tested positive for SARS-CoV-2 during March 1–April 7, 2020 and were not hospitalized (including outpatient and nonadmitted emergency department patients). During this time, the healthcare system operated a telephone triage line to manage patients with COVID-19–compatible symptoms. Patients with signs of severe illness (e.g., severe shortness of breath, confusion, or hemoptysis) were directed to the emergency department. Other symptomatic persons could receive outpatient SARS-CoV-2 testing through the healthcare system; because testing capacity was limited, appointments were prioritized for healthcare personnel and persons at high risk for severe illness, such as persons ≥65 years of age and those with underlying conditions, including diabetes mellitus, cardiovascular disease, and chronic respiratory disease. Telephone and telehealth follow-up calls were conducted for some patients. Trained personnel

reviewed information from electronic medical records (EMR) during April 7–May 15, 2020 on patient demographics, occupation, medications, underlying conditions, and symptoms using REDCap version 8.8.0 (https://projectredcap.org/software).

Analytic Methods

We categorized patients as having 0, 1, 2, or ≥3 of the following conditions: hypertension, diabetes mellitus, chronic kidney disease, chronic lung disease, HIV, chronic liver disease, history of organ transplant, cardiovascular disease, autoimmune or rheumatologic disease, obesity, and cancer. Obesity was defined as having a body mass index (BMI) ≥30. We considered insured patients to be those possessing private, Medicare, Medicaid, or other insurance policies. Race was classified as black or nonblack; data could not be disaggregated for other races or analyzed by ethnicity because of small sample sizes. We counted the number of classes of hypertension medication prescribed to patients (beta blockers, angiotensin-converting enzyme inhibitors, angiotensin receptor blockers, calcium channel blockers, thiazide diuretics, hydralazine, clonidine, or other); we analyzed combination medications by their individual components. Occupation was obtained from the EMR. Healthcare personnel (HCP) were defined as persons whose occupations included patient contact or possible exposure to infectious agents in the healthcare setting (*1*). Among the nonhospitalized group, care-seeking behavior was defined as having a COVID-19–related encounter with a healthcare provider documented in the medical record. We counted all healthcare encounters related to COVID-19 illness including doctor's office and urgent care visits (together classified as ambulatory care visits), emergency room visits, telehealth visits in which symptoms or care was reviewed, and calls to the telephone triage line. We compared characteristics and symptoms of hospitalized and nonhospitalized persons using X^2 or Fisher's exact tests for categorical variables; continuous variables are described with means and their standard deviations and compared with *t*-tests. We considered p values <0.05 to be significant.

Characteristics associated with hospitalization in this population have been described previously (*2*). We conducted further univariable and multivariable logistic regressions to explore the effects of additional age strata and multiple medical conditions (instead of individual conditions) on risk for hospitalization. The full multivariable logistic regression model included 6 age strata (18–29, 30–39, 40–49, 50–59, 60–69, and ≥70 years), number of underlying conditions (0, 1, 2, and ≥3), race, sex, insurance, and smoking (including current or former

smoking), as all of these characteristics were previously associated with hospitalization in this population (*2*). Firth's correction was used to account for small sample size in some groups (*3*). Because ≈50% of the nonhospitalized patients were HCP, possibly because of testing priorities in March–April 2020 (n = 168; 54%), we repeated multivariable models that excluded HCP as a sensitivity analysis.

We further explored the 3 most common medical conditions among the hospitalized and nonhospitalized populations: hypertension, diabetes, and obesity, all of which have been associated with increased risk of severe illness from COVID-19 (*4–8*). Diagnoses of hypertension and diabetes were identified from the medical history documented in the EMR; obesity was defined using calculations of BMI from weight and height recorded in the EMR. We investigated the effect of combinations of these 3 conditions, as well as degree of severity or control of these conditions, on risk for hospitalization. Among patients with hypertension, we investigated whether use of multiple classes of hypertension medication, considered a potential indicator of hypertension severity, was associated with hospitalization. Among patients with diabetes, we investigated the association of hemoglobin A1c (proportion of glycosylated hemoglobin in the blood tested within ≤1 year of when the medical records was reviewed for this study) and risk for hospitalization. Hemoglobin A1c levels were categorized as values <7% or ≥7%; this level was chosen as it is considered an indicator of adequate blood glucose control in patients with diabetes (*9*). Because of small sample size in the concurrent condition combination model (which tested for interactions between hypertension, diabetes, and obesity), we could not adjust for all previously identified risk factors. Therefore, we used confounding variables documented in the published literature, including: age (18–44, 45–64, and ≥65 years of age), race, HCP status, and the third comorbidity not used in the interaction term (*10–12*). Similarly, the models evaluating degree of control of concurrent conditions (BMI, number of antihypertensive medications, and hemoglobin A1c value) included age (18–44, 45–64, and ≥65 years of age), race, sex, HCP status, and hypertension (among patients with diabetes) and diagnosis of diabetes (all other models). We assessed additive interaction by calculating relative excess risk caused by interaction.

For all models, nonhospitalized patients were limited to those with a medical history and medication list documented in the EMR (n = 288). Multivariable models were limited to patients with complete data for all included variables; patients with missing data on any variable were

excluded from the analysis and the distribution and number of missing values were assumed to be random. All analyses were performed using SAS version 9.4 (SAS Institute Inc., https://www.sas.com). This activity was reviewed by the US Centers for Disease Control and Prevention. The study was conducted in accordance with applicable federal law and Centers for Disease Control and Prevention policy and by the Georgia Department of Public Health as an institutional review board–exempt public health evaluation.

References

1. Centers for Disease Control and Prevention. Interim infection prevention and control recommendations for healthcare personnel during the coronavirus disease 2019 (COVID-19) pandemic. 2020 [cited 2020 Nov 13]. https://www.cdc.gov/coronavirus/2019-ncov/hcp/infection-control-recommendations.html

2. Killerby ME, Link-Gelles R, Haight SC, Schrodt CA, England L, Gomes DJ, et al.; CDC COVID-19 Response Clinical Team. Characteristics associated with hospitalization among patients with COVID-19—metropolitan Atlanta, Georgia, March–April 2020. MMWR Morb Mortal Wkly Rep. 2020;69:790–4. PubMed https://doi.org/10.15585/mmwr.mm6925e1

3. Firth D. Bias reduction of maximum likelihood estimates. Biometrika. 1993;80:27–38. https://doi.org/10.1093/biomet/80.1.27

4. Adhikari SP, Meng S, Wu YJ, Mao YP, Ye RX, Wang QZ, et al. Epidemiology, causes, clinical manifestation and diagnosis, prevention and control of coronavirus disease (COVID-19) during the early outbreak period: a scoping review. Infect Dis Poverty. 2020;9:29. PubMed https://doi.org/10.1186/s40249-020-00646-x

5. Bialek S, Boundy E, Bowen V, Chow N, Cohn A, Dowling N, et al.; CDC COVID-19 Response Team. Severe outcomes among patients with coronavirus disease 2019 (COVID-19)—United States, February 12–March 16, 2020. MMWR Morb Mortal Wkly Rep. 2020;69:343–6. PubMed https://doi.org/10.15585/mmwr.mm6912e2

6. Richardson S, Hirsch JS, Narasimhan M, Crawford JM, McGinn T, Davidson KW, et al.; the Northwell COVID-19 Research Consortium. Presenting characteristics, comorbidities, and outcomes among 5700 patients hospitalized with COVID-19 in the New York City area. JAMA. 2020;323:2052–9. PubMed https://doi.org/10.1001/jama.2020.6775

7. Zhou F, Yu T, Du R, Fan G, Liu Y, Liu Z, et al. Clinical course and risk factors for mortality of adult inpatients with COVID-19 in Wuhan, China: a retrospective cohort study. Lancet. 2020;395:1054–62. PubMed https://doi.org/10.1016/S0140-6736(20)30566-3

8. Du RH, Liang LR, Yang CQ, Wang W, Cao TZ, Li M, et al. Predictors of mortality for patients with COVID-19 pneumonia caused by SARS-CoV-2: a prospective cohort study. Eur Respir J. 2020;55:2000524. PubMed https://doi.org/10.1183/13993003.00524-2020

9. Sherwani SI, Khan HA, Ekhzaimy A, Masood A, Sakharkar MK. Significance of HbA1c test in diagnosis and prognosis of diabetic patients. Biomark Insights. 2016;11:95–104. PubMed https://doi.org/10.4137/BMI.S38440

10. Rod JE, Oviedo-Trespalacios O, Cortes-Ramirez J. A brief-review of the risk factors for covid-19 severity. Rev Saude Publica. 2020;54:60. PubMed https://doi.org/10.11606/s1518-8787.2020054002481

11. Williamson EJ, Walker AJ, Bhaskaran K, Bacon S, Bates C, Morton CE, et al. Factors associated with COVID-19-related death using OpenSAFELY. Nature. 2020;584:430–6. PubMed https://doi.org/10.1038/s41586-020-2521-4

12. Bhargava A, Fukushima EA, Levine M, Zhao W, Tanveer F, Szpunar SM, et al. Predictors for severe COVID-19 infection. Clin Infect Dis. 2020;71:1962–8. PubMed https://doi.org/10.1093/cid/ciaa674

Appendix Table 1. Concurrent conditions among patients with coronavirus disease, Atlanta, Georgia, USA, 2020

Condition	Hospitalized, no. (%)* n = 220	Nonhospitalized, no. (%)* n = 311
Hypertension	142 (65)	101 (32)
Diabetes	81 (37)	30 (10)
Type I	2 (1)	2 (1)
Type II	74 (34)	28 (9)
Hemoglobin A1c, mean (SD)	8.1 (2.3)	6.8 (1.8)
Immunocompromising conditions	18 (8)	23 (7)
HIV/AIDS	5 (2)	10 (3)
Leukemia or lymphoma	1 (<1)	2 (1)
Solid organ or stem cell transplant	1 (<1)	0
Immunosuppressant use	12 (5)	11 (4)
Chronic kidney disease	38 (17)	7 (2)
End-stage renal disease	14 (6)	1 (<1)
Chronic lung disease	45 (20)	56 (18)
Asthma	22 (10)	40 (13)
Chronic obstructive pulmonary disease or emphysema	8 (4)	0
Obstructive sleep apnea	12 (5)	14 (5)
Interstitial lung disease	3 (1)	0
Sarcoidosis	0	3 (1)
Obesity		
BMI <30	86 (39)	123 (40)
BMI ≥30	124 (56)	104 (33)
Cardiovascular disease	56 (25)	36 (12)
Coronary artery disease	28 (13)	14 (5)
Cerebrovascular disease or stroke	5 (2)	4 (1)
Aortic regurgitation	7 (3)	9 (3)
Atrial fibrillation	12 (5)	9 (3)
Congestive heart failure	23 (10)	2 (1)
Other	10 (5)	14 (5)
Chronic liver disease	5 (2)	4 (1)
Alcoholic hepatitis	1 (<1)	0
Hepatitis B or C	3 (1)	0
Nonalcoholic fatty liver disease	1 (<1)	1 (<1)
Other	0	2 (1)

*Values are no. (%), except where indicated. BMI, body mass index.

Appendix Table 2. Characteristics of coronavirus disease patients with hypertension, diabetes, or obesity, Atlanta, Georgia, USA, 2020

Characteristic	Hospitalized, n (%)	Nonhospitalized, n (%)
Hypertension	142	101
Age, y		
18–29	0	3 (3)
30–39	8 (6)	12 (12)
40–49	12 (8)	18 (18)
50–59	27 (19)	31 (31)
60–69	44 (31)	24 (24)
≥70	51 (36)	13 (13)
Race		
Black	113 (80)	68 (67)
Nonblack	23 (16)	25 (25)
Missing data	6 (4)	8 (8)
Sex		
M	72 (51)	39 (39)
F	70 (49)	62 (61)
Insurance		
No	6 (4)	3 (3)
Yes	135 (95)	97 (96)
Missing data	1 (1)	1 (1)
Smoking		
Never	90 (63)	75 (74)
Current	8 (6)	3 (3)

Characteristic	Hospitalized, n (%)	Nonhospitalized, n (%)
Past	37 (26)	16 (16)
Missing data	7 (5)	7 (7)
Diabetes	81	30
Age, y		
18–29	1 (1)	2 (7)
30–39	7 (9)	4 (13)
40–49	5 (6)	3 (10)
50–59	18 (22)	9 (30)
60–69	23 (28)	9 (30)
≥70	27 (33)	3 (10)
Race		
Black	66 (81)	21 (70)
Nonblack	13 (16)	5 (17)
Missing data	2(2)	4 (13)
Sex		
M	42 (52)	10 (33)
F	39 (48)	20 (67)
Insurance		
No	7 (9)	1 (3)
Yes	74 (91)	29 (97)
Smoker		
Never	55 (68)	22 (73)
Current	2 (2)	1 (3)
Past	19 (23)	4 (13)
Missing data	5 (6)	3 (10)
Obesity	123	104
Age, y		
18–29	4 (3)	14 (13)
30–39	19 (15)	23 (22)
40–49	22 (18)	23 (22)
50–59	29 (24)	30 (29)
60–69	32 (26)	11 (11)
≥70	17 (14)	3 (3)
Race		
Black	103 (84)	67 (64)
Nonblack	16 (13)	23 (22)
Missing data	4 (3)	14 (13)
Sex		
M	55 (45)	29 (28)
F	68 (55)	75 (72)
Insurance		
No	13 (11)	6 (6)
Yes	109 (89)	96 (92)
Missing data	1 (1)	2 (2)
Smoker		
Never	88 (72)	86 (83)
Current	8 (7)	1 (1)
Past	22 (18)	13 (13)
Missing data	5 (4)	4 (4)

Appendix Table 3. Detailed characteristics of hospitalized and nonhospitalized patients with coronavirus disease, Atlanta, Georgia, USA, 2020*

Characteristic	Patients, no. (%) Total hospitalized n = 220	Total nonhospitalized n = 311	Hospitalized, non-HCP n = 212	Nonhospitalized, non-HCP n = 143
Age, y				
18–29	5 (2)	52 (17)	3 (1)	16 (11)
30–39	24 (11)	79 (25)	23 (11)	22 (15)
40–49	36 (16)	54 (17)	35 (17)	24 (17)
50–59	41 (19)	63 (20)	39 (18)	33 (23)
60–69	56 (25)	41 (13)	55 (26)	27 (19)
≥70	58 (26)	22 (7)	57 (27)	21 (15)
Race				
Black	174 (79)	139 (45)	168 (79)	70 (49)
Nonblack	36 (16)	100 (32)	34 (16)	53 (37)
Missing data	10 (5)	72 (23)	10 (5)	20 (14)
Sex				
F	106 (48)	197 (63)	101 (48)	72 (50)
M	114 (52)	114 (37)	111 (52)	71 (50)
Insurance				
No	22 (10)	20 (6)	21 (10)	8 (6)
Yes	195 (89)	285 (92)	188 (89)	133 (93)
Missing data	3 (1)	6 (2)	3 (1)	2 (1)
Smoker				
Nonsmoker	157 (71)	230 (74)	151 (71)	105 (73)
Smoker	54 (25)	37 (12)	52 (25)	24 (17)
Missing data	9 (4)	44 (14)	9 (4)	14 (10)
No. concurrent conditions				
0	21 (10)	122 (39)	20 (9)	44 (31)
1	48 (22)	80 (26)	46 (22)	38 (27)
2	71 (32)	68 (22)	69 (33)	38 (27)
≥3	80 (36)	41 (13)	77 (36)	23 (16)

*HCP, healthcare personnel.

Appendix Table 4. Multiple concurrent conditions among coronavirus disease patients, Atlanta, Georgia, USA, 2020*

Conditions	Patients, n (%) Hospitalized (n = 220)	Nonhospitalized (n = 288)	Estimate†	SE	p value	RERI (95% CI)
Hypertension and diabetes						
Intercept			−0.8952	0.3345	<0.01	0.08 (−4.09 to 4.26)
Hypertension	77 (35)	72 (25)	0.2496	0.3118	0.64	
Diabetes	16 (7)	6 (2)	1.1662	0.5804	0.04	
Hypertension + diabetes	65 (30)	19 (7)	−0.1448	0.6974	0.84	
Diabetes and obesity						
Intercept			−0.9403	0.3392	<0.01	0.34 (−4.80 to 5.48)
Obesity	70 (32)	81 (28)	0.8239	0.2982	<0.01	
Diabetes	25 (11)	8 (3)	1.3913	0.5128	<0.01	
Diabetes + obesity	53 (24)	13 (5)	−0.4829	0.6533	0.46	
Hypertension and obesity						
Intercept			−1.0593	0.3550	<0.01	−1.36 (−3.79 to 1.07)
Hypertension	53 (24)	29 (10)	0.6455	0.3944	0.10	
Obesity	41 (19)	46 (16)	1.0671	0.3700	<0.01	
Hypertension + obesity	82 (37)	48 (17)	−0.8162	0.5130	0.11	

*RERI: relative excess risk due to interaction; SE, standard error.
†Adjusted for age, race, healthcare personnel status, and other concurrent conditions.
